# The Effect of a Life-Stage Based Intervention on Depression in Youth Living with HIV in Kenya and Uganda: Results from the SEARCH-Youth Trial

**DOI:** 10.3390/tropicalmed10020055

**Published:** 2025-02-14

**Authors:** Florence Mwangwa, Jason Johnson-Peretz, James Peng, Laura B. Balzer, Janice Litunya, Janet Nakigudde, Douglas Black, Lawrence Owino, Cecilia Akatukwasa, Anjeline Onyango, Fredrick Atwine, Titus O. Arunga, James Ayieko, Moses R. Kamya, Diane Havlir, Carol S. Camlin, Theodore Ruel

**Affiliations:** 1 Infectious Diseases Research Collaboration, Kampala P.O. Box 7074, Uganda; 2 University of California, San Francisco, San Francisco, CA 94158, USA; 3 Department of Biostatistics, University of Washington, Seattle, WA 98195, USA; 4 Division of Biostatistics, School of Public Health, University of California, Berkeley, CA 94720, USA; laura.balzer@berkeley.edu; 5 Kenya Medical Research Institute, Njoro P.O. Box 517-20107, Kenya; 6 Department of Psychiatry, College of Health Sciences, Makerere University, Kampala P.O. Box 7072, Uganda; janetnakigudde@gmail.com; 7 Kenya Medical Research Institute, Kisumu P.O. Box 614-40100, Kenya

**Keywords:** depression, HIV, young adults, adolescents, sub-Saharan Africa

## Abstract

Depression among adolescents and young adults with HIV affects both their wellbeing and clinical care outcomes. Integrated care models are needed. We hypothesized that the SEARCH-Youth intervention, a life-stage-based care model that improved viral suppression, would reduce depressive symptoms as compared to the standard of care. We conducted a mixed-methods study of youth with HIV aged 15–24 years in SEARCH-Youth, a cluster-randomized trial in rural Uganda and Kenya (NCT03848728). Depression was assessed cross-sectionally with the PHQ-9 screening tool and compared by arm using targeted minimum loss-based estimation. In-depth semi-structured interviews with young participants, family members, and providers were analyzed using a modified framework of select codes pertaining to depression. We surveyed 1,234 participants (median age 21 years, 80% female). Having any depressive symptoms was less common in the intervention arm (53%) compared to the control (73%), representing a 28% risk reduction (risk ratio: 0.72; CI: 0.59–0.89). Predictors of at least mild depression included pressure to have sex, physical threats, and recent major life events. Longitudinal qualitative research among 113 participants found that supportive counseling from providers helped patients build confidence and coping skills. Integrated models of care that address social threats, adverse life events, and social support can be used to reduce depression among adolescents and young adults with HIV.

## 1. Introduction

Adolescents and young adults living with HIV (AYAH) in sub-Saharan Africa bear a disproportionate burden of HIV and experience high rates of depression [1,2]. Nearly half of the world’s youth aged 15–24 years living with HIV reside in eastern and southern Africa, estimated to number 1.9 million, including 160,000 new infections in 2023 [3]. Mental health disorders are prevalent in this population and have significant health impacts affecting engagement with HIV care and overall wellbeing [4,5,6]. In Kenya and Uganda, an estimated 50% or more AYAH experience symptoms of depression, with 5–18% meeting the criteria for major depression in some settings [7,8,9,10,11].

For AYAH in sub-Saharan Africa, changes in social support structures, adverse life events, and social stressors related to HIV infection have a complex and synergistic negative overlap, affecting HIV-related mental health outcomes [12,13,14]. HIV-associated stigma has been associated with depressive symptoms, nonadherence to treatment, and detectable viral loads among AYAH in western Kenya [15]. In a study from rural Uganda, 16% of adolescents with HIV reported suicidal ideation in the prior month [7], and 17% of a cohort of Ethiopian young adults reported prior suicide attempts [16]. Low self-esteem among youth with HIV is associated with viral non-suppression [17]. A systematic review of mental disorders in AYAH in sub-Saharan Africa found that female sex, bullying, HIV-related stigma, low social support, and poor antiretroviral therapy adherence were associated with depression [14]. Self-reported poor health and negative life events were associated with depression in a study of youth with HIV aged 8–17 years in Zambia [18]. Death within the family and advanced HIV were associated with suicidal ideation among AYAH in Ethiopia [19].

While evidence supports some mental health interventions for youth in sub-Saharan Africa, the synergistic and overlapping burden of HIV and depression in people living with HIV highlights the need for integrated solutions [20,21] Interventions that focus on family-strengthening and parent–child relationships have shown promising results in improving mental health in adolescents with HIV in low- and middle-income countries, with some success from group- and school-based counseling [22]. The *SUUBI + Adherence*, a family economic strengthening intervention, showed reductions in symptoms of hopelessness, but the benefit declined after some time and the intervention did not significantly impact symptoms of depression [23].

Few intervention studies integrate mental health and HIV care for AYAH in this region [24]. The *Sauti ya Vijana,* a group mental health intervention targeting youth and their caregivers, showed promising results in virologic suppression in a pilot study [25]. *Zvandiri*, another intervention, was a multi-component-differentiated service delivery model that integrated community- and clinic-based health services with psychosocial support within government health services in Zambia [26]. *Zvandiri* was found to increase virologic suppression in adolescents and showed a trend of reducing the prevalence of depression [27]. The addition of peer-delivered problem-solving therapy to *Zvandiri* was subsequently found to reduce the prevalence of anxiety and depression among adolescents [28].

With the SEARCH-Youth multi-level health system intervention, we sought to address the social and psychological dynamics affecting AYAH in rural Uganda and Kenya. The intervention had four components: (1) a life-stage based assessment tool, including questions about recent events, social support, HIV disclosure, resources, mental health, and substance use; (2) a solution-oriented discussion between the provider and client around alternative access to the clinic to address barriers to care and adherence; (3) rapid viral load feedback with results obtained and discussed within 72 h; and (4) a secure chat network for providers to discuss challenging cases. We evaluated the intervention in a cluster-randomized, open-label trial and found a 10% relative increase in the prevalence of virologic suppression after 2 years [29].

The purpose of this study was to determine if the SEARCH-Youth intervention reduced depression among AYAH, by comparing the prevalence of depressive symptoms between the intervention and control arms. To understand the intervention’s potential mechanisms of action, we also evaluated predictors of depression and analyzed qualitative data to explore how youth with HIV navigated mental health-related challenges.

## 2. Materials and Methods

### 2.1. Study Context

SEARCH-Youth (NCT03848728) was a cluster-randomized trial of AYAH aged 15–24 years and receiving care in 28 rural clinics in western Kenya and southwestern Uganda [29]. Community public health clinics were pair-matched and randomized to the intervention and control arms. SEARCH-Youth enrolled participants between 14 March 2019 and 26 November 2020. As previously described [29], sample size and power calculations were conducted for the trial’s primary outcome, which was HIV viral suppression and determined after 2 years of follow-up. Depression was a secondary outcome that was determined at the study closure. Participants in both arms received all locally implemented youth programs and the Ministry of Health recommended adherence and psychosocial interventions, including peer support. Participants in the intervention arm additionally received the SEARCH-Youth intervention every three months. The full details of the SEARCH-Youth intervention are reported elsewhere [29].

### 2.2. Depression Survey—Participants

SEARCH-Youth participants who had been assessed for the primary endpoint in both study arms were eligible for the depression survey. Eligible participants were approached for consent to participate in the depression survey between January and June 2023, during the final year of the trial.

### 2.3. Depression Survey—Data Collection

Demographic data from the time of enrolment into the SEARCH-Youth trial included country, sex, age, gender, and antiretroviral therapy (ART) status. We classified ART status as “new to care” (started ART within the prior 6 months or at enrolment), “already in care” (started ART more than 6 months ago with a clinic visit in the prior 6 months), and “re-engaging in care” (started ART more than 6 months ago but did not have a clinic visit in the prior 6 months). Participants who consented to the depression study were administered a survey that included the 9-item Patient Health Questionnaire (PHQ-9) (Table A1, Appendix A). The PHQ-9 scores the presence of the 9 DSM-IV (Diagnostic and Statistical Manual of Mental Disorders, 5th ed.) [30] depression criteria over the preceding 14 days on a 4-point Likert scale from 0 (not at all) to 3 (every day). The PHQ-9 has been evaluated for use in the local languages of our sites, including Swahili [31], DhoLuo [32], Luganda [33], and Runyankore [34], and specifically for use with people living with HIV [35]. The PHQ-9 was supplemented by questions about substance use, major life events in the prior 3 months, pregnancies, feeling supported, history of threat of physical harm, and feeling pressured to perform sexual activity (Table A1, Appendix A). The survey questionnaire was adapted with the guidance of a child and adolescent psychologist, translated into the local languages, and administered by trained study clinicians.

### 2.4. Depression Survey—Analysis

To evaluate the effect of the SEARCH-Youth intervention on depressive symptoms, we estimated the prevalence of 3 outcomes: “any depressive symptoms” was a PHQ-9 score ≥ 1, “at least mild” depression was a PHQ-9 score ≥ 5, and moderate/severe depression was a PHQ-9 score ≥ 10. We compared the risk of each outcome between arms using a two-stage targeted minimum loss-based estimator (TMLE) accounting for clustering and with adaptive adjustment to improve precision (country, age, sex in the candidate adjustment set) [36]. We created 95% confidence intervals and tested the null hypothesis that the SEARCH-Youth intervention did not reduce depression with a one-sided test at the 5% significance level. We also evaluated effectiveness within pre-specified subgroups defined by sex, age (15–19 years versus 20–24 years), and ART status. Pooling over arms and within each arm separately, we assessed predictors of at least mild depression with multivariable logistic regression adjusted for age, sex, and country. Predictors were collected at the time of the survey and included major life events in the prior three months as well as measures of social support and social pressure.

### 2.5. Qualitative Study—Participants

The longitudinal qualitative component followed a subset of participants (n = 113) for three years, to identify factors in participants’ lives and experiences with the intervention that influenced engagement (or re-engagement) in HIV care. We systematically selected AYAH from eight communities, balanced on country and with attention to the trial arm, sex, age (15–17 vs. 18–24 years), and ART status. We also interviewed family members chosen by participants as being involved in their care (n = 60). Within the intervention arm only, we interviewed healthcare providers (n = 38) from these community clinics.

### 2.6. Qualitative Study—Data Collection

Native speakers of the local languages DhoLuo, Runyankole, Swahili, and English from a gender-balanced team of trained qualitative researchers (2 female, 3 male: CA, AO; LO, FA, TOA) conducted audio-recorded semi-structured interviews in private locations comfortable for the participants (e.g., private houses or clinic rooms) to maintain confidentiality. All participants provided written and informed consent for the interviews to be audio-recorded and transcribed. The interviews explored participant experiences with HIV diagnosis and care history, ART adherence, social support systems, HIV status disclosure, and other contextual life elements following guides designed by a senior author (CSC) with contributions from the full qualitative team (CA, AO, LO, FA, TOA, MG, JL).

We conducted interviews with SEARCH-Youth participants at three time points: baseline (June to December 2019; n = 83); year 1 (September 2020 through February 2021; n = 76, of whom 27 were new to replace prior participants who had transferred or moved away); and year 2 (October through December 2021; n = 46, all among intervention participants previously interviewed). We interviewed family members and providers either at baseline (June 2019 to December 2019) or during year 1 (October 2020 to March 2021).

Following the interviews, the team translated and transcribed them into English. The seven-person team (LO, AO, CA, FA, IM, JL, JJP) then uploaded these transcripts to Dedoose and coded the data using a codebook incorporating both *a priori* and emergent codes. The theory-informed *a priori* codes provided the basis of broad codes, while a full-group review (with CSC) of an early set of transcripts added inductively derived child codes reflecting phenomena and concepts present in the data.

### 2.7. Qualitative Study—Analysis

The co-first author (JJP) undertook a separate analysis of the coded interviews after quantitative analysis revealed the potential effects of life-stage assessments on depression among AYAH. This analysis sought to uncover the factors, as reported by the participants, and pathways, as interpreted by the researchers, that might have impacted depression in this cohort.

From an initial set of ten codes, the co-first author (JJP) selected four: Mental Health, Other Support, Adolescent-Focused Care, and Provider Training. These codes underwent framework analysis using Excel tables, which prompted the further inclusion of excerpts containing keywords that did not have independent, associated codes: life stage; depression; hate; and suicid* (=suicide and suicidal). A final analysis prompted the re-incorporation of a final code (Acceptance of Status), which was among the initial ten codes. The completed analysis of potential mechanisms applicable to depression and life-stage assessments was then compiled and arranged thematically for presentation.

This article is a revised and expanded version of results presented at the Conference on Retroviruses and Opportunistic Infections [Denver, Colorado, USA, 3–6 March 2024] [37].

## 3. Results

### 3.1. Participants

**Figure 1:**
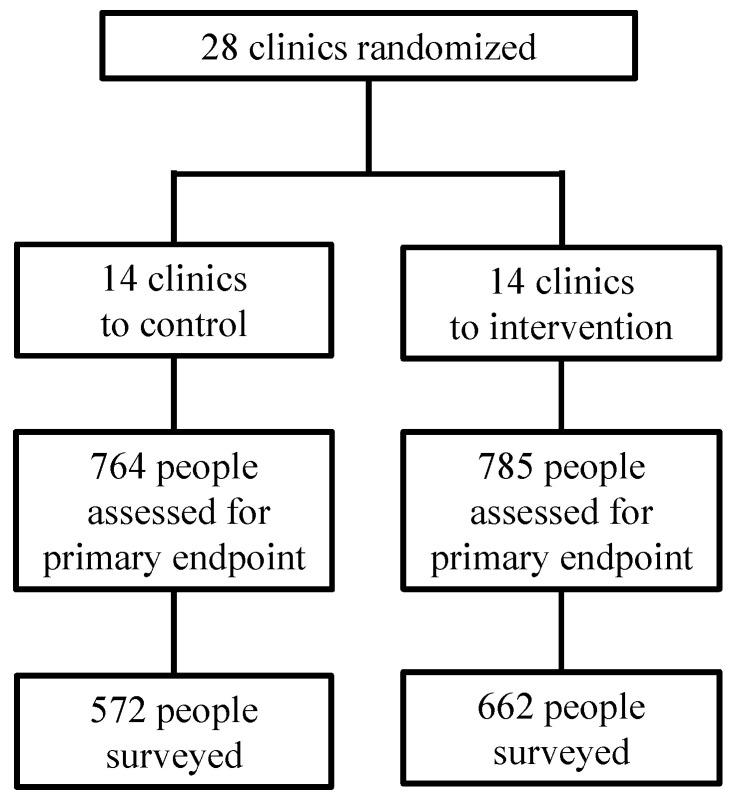
Participants included in the depression study.

Among the 1988 participants enrolled in the SEARCH-Youth trial, 1549 were included in the primary endpoint analysis and eligible for participation in the depression survey [29]. Of those, we reached and interviewed a total of 1234 (80%) for the survey, including 572 in the control and 662 in the intervention arm (Figure 1). Baseline characteristics of the survey participants were balanced by arm (Table 1). The median age was 21 years; 80% of the participants were female; 65% had completed primary school; 30% were new to or re-engaging with care; and 54% were from Uganda. The participants were surveyed after a median of 3.76 (IQR: 3.64–3.93) years in the intervention arm and 3.75 years (3.65–3.86) years in the control arm. The characteristics of the participants (n = 113) in the qualitative study were similar to those of the quantitative survey (overall in Table 1 and by arm in Table A2, Appendix A).

#### 3.1.1. Prevalence of Depression

**Figure 2:**
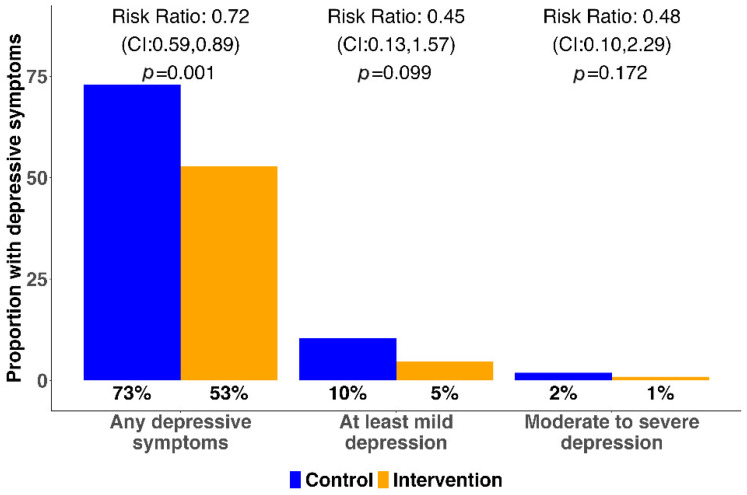
Effectiveness of the SEARCH-Youth intervention on depression.

The proportion of participants reporting any symptoms of depression (PHQ-9 score ≥ 1) was 52.8% in the intervention arm as compared to 73.0% in the control arm, representing a 28% risk reduction (RR: 0.72; 95% CI: 0.59–0.89; *p* = 0.001; Figure 2). The prevalence of at least mild depression (PHQ-9 ≥ 5) was 4.7% in the intervention as compared to 10.5% in the control, corresponding to a 55% risk reduction (RR: 0.45; 95% CI: 0.13–1.57; *p* = 0.099). The risk of having moderate to severe depression (PHQ-9 ≥ 10) was low in both the intervention and control arms (0.9% versus 1.9%, respectively) and 52% lower among the intervention participants (RR: 0.48; 95% CI: 0.10–2.29; *p* = 0.172). Additional summary measures of scores on the PHQ-9 survey are found in Table A3 (Appendix A).

**Figure A1:**
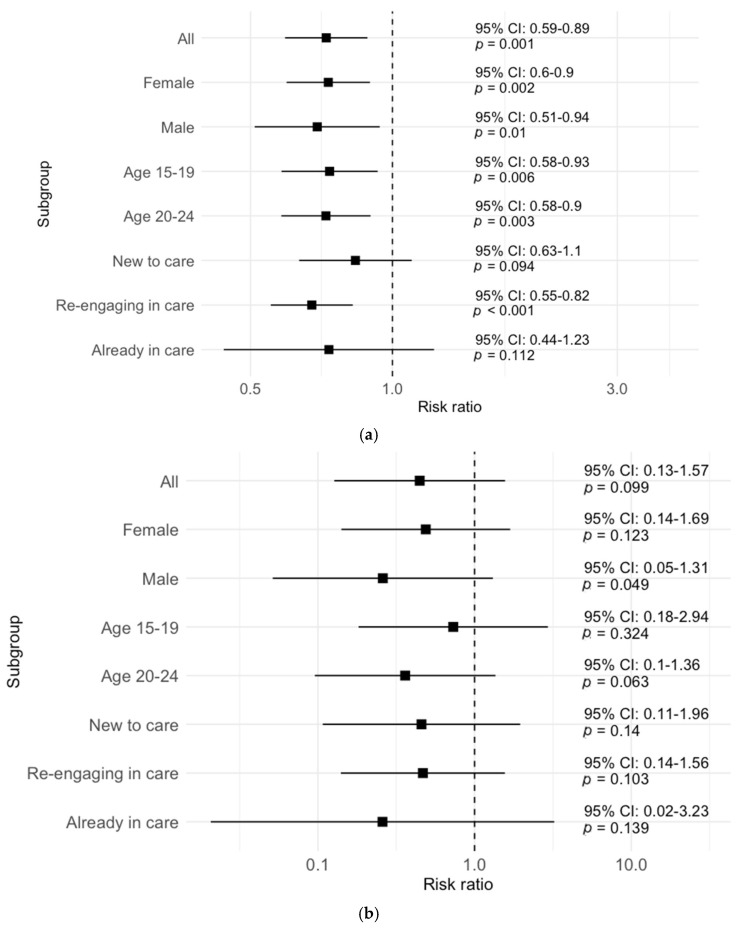

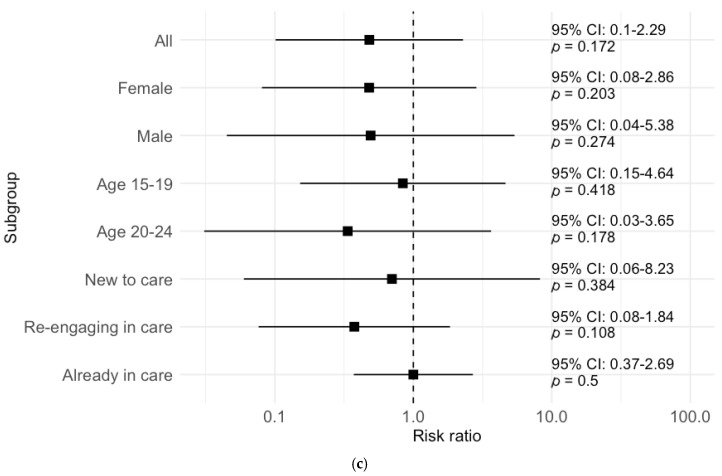
Intervention effectiveness on depressive symptoms by subgroup. Each graph shows the point estimate and 95% confidence for the risk ratio (intervention relative to control). The dashed vertical line is the null value (risk ratio = 1). (**a**) Risk ratios for any symptoms of depression by subgroup. (**b**) Risk ratios for symptoms indicating at least mild depression by subgroup. (**c**) Risk ratios for symptoms indicating moderate to severe depression by subgroup.

The risk of having any symptoms of depression (PHQ-9 ≥ 1) was lower in the intervention arm than the control arm within sub-groups defined by sex, age, and ART status. As compared to the control participants, the risk was 27% lower among females (RR: 0.73; 95% CI: 0.60–0.90; *p* = 0.002) and 31% lower among males (RR: 0.69; 95% CI: 0.51–0.94; *p* = 0.010) in the intervention arm (Figure A1, Appendix A). Similar effects were observed across the age groups of 15–19 years (RR: 0.74; 95% CI: 0.58–0.93; *p* = 0.006) and 20–24 years (RR: 0.72; 95% CI: 0.58–0.90; *p* = 0.003). Among the strata of ART status at baseline, the largest effect size was among participants re-engaging with care; the prevalence of any symptoms of depression was 33% lower among intervention participants than control participants (RR: 0.67; 95% CI: 0.55–0.82; *p* < 0.001). The relative risk was lower but not statistically significant among those newly initiating care (RR: 0.73; 95% CI: 0.44–1.23; *p* = 0.112) and those already in care at baseline (RR: 0.83; 95% CI: 0.63–1.10; *p* = 0.094). Subgroup patterns were similar for at least mild (PHQ-9 ≥ 5) and moderate to severe depression (PHQ-9 ≥ 10), but with fewer affected participants in both arms, the contrast did not reach statistical significance. (See Figure A1 in Appendix A).

Suicidal thoughts of any frequency occurred in 2.2% (27/1234) of participants and was more common in control participants, as compared to intervention participants (Table A3, in Appendix A). Specifically, “Thoughts that you would be better off dead” was reported as occurring on several days among eleven (1.9%) control participants and seven (1.1%) intervention participants. These thoughts occurred on more than half the days among six (1.0%) control participants and two (0.3%) intervention participants. The only participant who had these thoughts nearly every day was in the intervention arm. Participants who reported any suicidal thoughts were referred for counseling and followed up closely.

#### 3.1.2. Prevalence of Recent Major Life Events

Major events in the 3 months preceding the survey were reported by 452 (36.5%) participants, with a greater proportion in the control (40.6%) than in the intervention arm (33.1%) (Table 2). The most common event was a change of residence and reported by a slightly greater proportion of control participants than intervention participants (17.3% vs. 10.9%). Family strife was more prevalent in the control arm (2.4% versus 1.1%), but relationship strife was less common (2.4% versus 3.8%).

#### 3.1.3. Social Pressures, Social Supports, and Substance Use

More participants in the control arm than in the intervention arm did not feel supported by the people around them (5.6% versus 2.6%), reported feeling physically threatened (4.9% versus 3.0%), and felt pressured to have sex (2.3% versus 0.5%). The same proportion reported drinking alcohol in the two arms (19.2% versus 19.5%), and very few (<2%) reported using other substances (Table 3).

#### 3.1.4. Predictors of at Least Mild Depression

**Figure 3:**
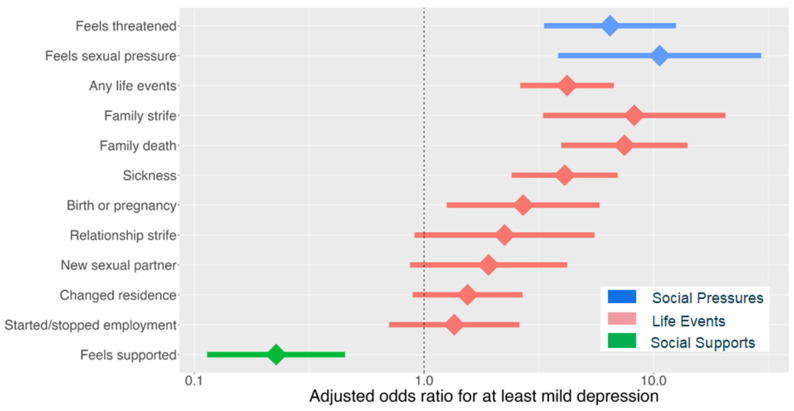
Predictors of at least mild depression.

Our second objective was to identify the predictors of having at least mild depression (PHQ-9 score ≥ 5) [38] in our study population. We found that feeling pressured to have sex (adjusted odds ratio (aOR): 10.6; 95% CI: 3.8–29.4), feeling physically threatened (aOR: 6.5; 95% CI: 3.3–12.5), and having experienced recent significant life events (aOR: 4.2; 95% CI: 2.6–6.7), including physical sickness (aOR: 4.1; 95% CI: 2.4–7.0), family death (aOR: 7.5; 95% CI: 4.0–14.1), and birth or recent pregnancy (aOR: 2.7; 95% CI: 1.3–5.8) were associated with at least mild depression, controlling for age, sex, and country (Figure 3). In contrast, feeling supported was associated with lower odds of having at least mild depression (aOR: 0.23; 95% CI: 0.11–0.45).

### 3.2. Qualitative Results

In the qualitative cohort, participants described several negative mental health consequences of living with HIV that they explicitly noted were alleviated through provider interactions. We present those examples first before examining how provider counseling may have led to greater self-acceptance, as one potential mechanism through which the SEARCH-Youth intervention reduced depressive symptoms. We close with a brief overview of providers’ perceptions about the life-stage assessment to highlight how this tool may have facilitated provider counseling.

#### 3.2.1. Depression: Social Etiologies

Participants specified three specific types of negative feelings that the intervention may have helped alleviate: suicidal thoughts, loneliness, and self-hate.

##### Suicidal Thoughts

Suicidal thoughts were a common response to stress, frustration, or diagnosis and could also be prompted by fear of a spouse leaving or fear of never being able to get married because of one’s HIV status. One participant expressed it this way: *“It is true that I wanted to commit suicide because I lost hope in life. By then, I was not married yet; hence, I was thinking, how will I get married?”* (Male, 24 y.o., intervention arm, Kenya).

Not wanting to be on HIV medication led another participant towards a suicide attempt using her mother’s antiretroviral medications. In this case, the providers spoke to the youth participant about “the goodness of being on HIV medication”, which led her to self-acceptance of her status:

“I had just tested positive, and I never wanted to take HIV drugs, that’s why I wanted to kill myself. What saved me was the people at home who came to my rescue when I was still vomiting, they gave me water and porridge, and I became okay. After some days I was taken to the health facility where counsellors educated me about the goodness of being on HIV medication. That’s when I accepted to start care, and by the way, I was able to regain my energy thereafter”.

(*Female, 23 y.o., intervention arm, Uganda*)

Similarly, an inability to discuss one’s feelings or problems led another youth participant to attempt overdosing on his antiretroviral medications, as his wife described:

“People always conflict when staying together, and so instead of sitting down and talking about the issue he just takes an overdose of the medication; he always believes that is the only solution. <Later in the interview> I think that is what runs through his mind <that I will leave him> but I told him that leaving would not solve anything and if I were to leave, I would not have come back after I left. The provider is the one who called and convinced me to come back, so we came to the same provider who counselled him, and he promised that he had understood. … [But] it did not even take a month before he took another overdose”.

(*Spouse of 23 y.o. male, control arm, Kenya*)

In this instance, provider counseling seemed to alleviate feelings of suicidal ideation for a time and broker peace between parties in the participant’s life. Provider counseling importantly included advising other family members, as one spouse admitted: “*We were counselled as a couple, and because of the provider’s advice, I was able to understand and stay married to him despite his status. He might have committed suicide if I had left him*”. (Spouse of 24 y.o. male, control arm, Kenya).

Despite the variation in reasons for wanting to end one’s life, provider discussions, prompted in part by the life-stage assessments, seem to have encouraged these participants to talk about their circumstances, led providers to counsel other family members, and increased feelings of support among AYAH.

##### Loneliness

Feelings of loneliness, or, specifically, of being the only young person living with HIV, were pervasive in our cohort. As one family member noted, “*The other issue is that he is the only child in my village known to be HIV positive. He started to feel like he was the only one affected and he started to hate himself*”. (Paternal grandmother of 16 y.o. male, control arm, Uganda). Importantly, outside the life-stage assessments, the sense of isolation was alleviated by seeing fellow youth at the clinic.

“I did not feel good at that time, I hated myself inside me. I asked myself, at this age of mine, I am HIV positive, what is my future going to be like? I had so many thoughts. After I started coming here to the clinic, I would meet young people, those who are breast feeding, and those who are older than me. I saw my age-mates; I saw that everyone is affected, and I accepted my situation the way it was. I believed that HIV does not kill, it only kills fools”.

(*Female, 18 y.o., intervention arm, Uganda*)

In a similar vein, one provider attributed one youth’s viral non-suppression to “*the issue of neglect because she is a total orphan. So she feels there is no need of using those drugs*”. (Clinical Officer, intervention arm, Kenya). The life-stage assessment prompted healthcare providers to refer those AYAH to peer counsellors or case managers for follow-up with additional support, especially around medication adherence.

##### Self-Hatred

Another driver of depressive symptoms was self-hatred, stemming from fear of rejection, a sense of being alone, or legitimate fear of being killed by someone because of their gender, HIV status, or the intersection of the two. As one participant succinctly put it, *“I started thinking about committing suicide because I knew people would kill me after getting to know that I had HIV”.* (Female, 19 y.o., intervention arm, Uganda). Like with loneliness as above, self-hatred due to being alone often disappeared with recognition that they were not alone.

For perinatally infected youth, self-hate was also tied to the age of infection, as one young woman explained: “*I still think the virus came from my parents because when I tested positive, I almost committed suicide. That’s when my mother consoled me by sharing to me how I got HIV from her*”. (Female, 23 y.o., intervention arm, Uganda). Another youth related his self-hatred to confusion about why he was positive and his siblings were not and the role that providers had in attenuating that self-hatred:

“I have been motivated through pieces of advice and health education from the providers as well as family members. They always try to offer me counselling and advise me because they would not want to lose me. Therefore, I just adhere to my medication for the sake of my family but to me, I hate it. … I do not understand why I was born with the virus. This has been my concern ever since. My younger brother, who passed on, was not infected; yet the first-born is infected, and I do not know why. … The second born and third born are not infected; hence, I cannot understand why it is so”.

(*Male, 18 y.o., intervention arm, Kenya*)

He was not alone in these feelings. Many AYAH said that self-hate is part of the process of moving towards self-acceptance and providers helped that process along: “*I don’t think there’s anything that can remove the enacted stigma; all youths have to first experience self-hate after testing HIV positive*”. (Female, 22 y.o., intervention arm, Uganda).

Self-hate was sometimes also tied to others discussing AYAH’s health status, making them self-conscious at an age when self-consciousness is a powerful feeling. Likewise, self-hate could stem from fear of others finding their ART, rather than finding out about their HIV status per se.

“I was thinking to myself, if people get to learn that I am on ART—I am the last born of five girls—if they get to learn that I am HIV positive, won’t they reject me? But later, it did not matter to me whether they rejected me; I just decided to take my ARVs—you never know what the future holds. … Later on, I realized that most of the people at the HIV clinic were from my village, and I also felt strongly that I was not alone in this situation”. (ARV = antiretroviral medication).

(*Female, 22 y.o., intervention arm, Uganda*)

AYAH also mentioned self-hate being tied to thoughts of early death or to being in a liminal state between “healthy life” and death, a situation resulting when people do not die immediately of HIV and they feel stuck, neither “here” nor “there”.

“The issue I had with S is that he hated himself; he suffered self-rejection, but ‘death’ also rejected him, and his situation was so bad. He was not like a human being, S consumed a lot of alcohol to the extent that sometimes we had to carry him off the road. When he would get home, he would say, ‘If only it was possible, I would take poison and die’. … I was bothered about the fact that he is a young person that hates himself. But now I am happy that at this point in time, he loves himself and his partner as well. I ask God that S stays with his wife”.

(*Sister of 23 y.o. male, control arm, Uganda*)

#### 3.2.2. Self-Acceptance

In contrast to self-hate, AYAH mentioned several factors leading to self-acceptance, many brokered by the counseling portion of the intervention. These included providers encouraging youth to continue taking their medication: *“Personally, I thought of defaulting from taking the drugs because this disease is very bad, and I could not wish to be infected. However, he encouraged me that if I just continue adhering to my medication. I will be okay”.* (Male, 16 y.o., intervention arm, Kenya).

Other youth mentioned feeling supported by both family and home visits from counsellors and, crucially, by the family itself supporting the home visits:

“It happened that she visited the hospital for her antenatal services and there she [my wife] got tested HIV positive. She immediately called me, and I did not show up but instead invited the nurse to our home a few days later. The nurse visited, and we had very good times together full of counselling. That night after the nurse, we also talked about our own life and how to live and we basically accepted the condition. I assured her that despite not being HIV positive, I am ready to live with her”.

(*Husband of 20 y.o. female, intervention arm, Kenya*)

The AYAH also noted that becoming less self-conscious helped. This was fostered by no longer feeling alone after realizing how many people in the community have HIV. *“I really felt bad. I actually thought of jumping into a stream and killing myself, but with time, I realized many people in my community were HIV-positive and in care. So after some time, my heart relaxed”.* (Female, 16 y.o., intervention arm, Uganda).

Importantly, acceptance sometimes preceded enrolment in care; in these cases, the intervention functioned as a support for structures already in place at home. For example, family members mentioned how providers (who were sometimes also relatives) counseled them on how to handle AYAH: “*Other days he <my son> would call and tell me he was sick and I told my sister-in-law about it. She told me to handle my son gently and encourage my son to come back so that we could help him. ‘If you handle him harshly, he will feel rejected because of his status.’*”

(*Mother of 24 y.o. male, intervention arm, Kenya*)

#### 3.2.3. Life-Stage-Based Assessment, from the Provider’s Perspective

Intervention arm providers described how the life-stage assessments functioned as an organized way to find out what was going on in the AYAH’s lives and uncover barriers to care. They characterized the assessments as a process of working with young people as they grew emotionally and began to open up about their challenges at home and school. This was aided by the fact that life-stage assessments could be performed at the facility, during a home visit, or over the phone.

“In the past, if [AYAH] had an issue, they would deal with it personally … Currently, if they come, we start by asking them about their issues before we even open the files. We talk to them about general issues not regarding their medication. I believe that SEARCH Youth brought that aspect and created time for talking about their issues without worrying about reducing the queue. Most of the clients feel that this is the best thing that happened to them. Most of them would call and write messages asking if I am at the clinic. If I tell them I am not around, they will not come because they believe that they need to talk”.

(*Nurse, intervention arm, Kenya*)

Some providers also noted that life-stage counseling for adolescents is different from that for young adults who are married and benefit from couples’ counseling. For the providers, these conversations and the social connectedness they fostered were key to encouraging young people to open up and share their challenges. As one provider summarized, *“I had never been in a situation where I had to deal with youth most of the time. I think I have learnt as a study clinician, and now I am able to understand the youths. [Now] I know that they need to be handled cautiously and that has increased my work experience. It is like specializing in something*”. (Clinical Officer, intervention arm, Kenya). Other providers concurred:

“Another thing I have learnt is if a youth does not like you, the health provider, you cannot offer them HIV care at all. …Yes, you must build a rapport. Those youth rarely give their trust to anyone; if they, for instance, have not liked you, they won’t share with you anything, and actually they might not keep you as their provider. It’s better to refer them to someone else they are free with and whom they like”.

(*Clinical Officer, intervention arm, Uganda*)

“For them to trust me, they discovered that I would keep their secrets…. Those already in care always refer their friends to me in case they need any HIV care services, after assuring them that I would confidentially keep their health information”.

(*Peer Educator 4, intervention arm, Uganda*)

In fact, provider counseling was supported by prior training as well as the SEARCH-Youth training. One provider in Uganda mentioned specific training on depression. Two providers in Kenya mentioned mentorship on adolescent issues, while a third performed self-training via reading. Importantly, one of them referenced how the training attuned them to sexual violence among adolescents:

“The things that adolescents don’t like and how these can be communicated to them? For example, the adolescent may come, and they have been raped, you ask them, and they cannot give you a response. But when you observe, you notice that the way she is sitting is not right…the sitting posture. How to handle them when they have a challenge, getting them treatment buddies. You link them to a fellow youth and let them know that this one is in the same situation as they are”.

(*Peer educator 2, intervention arm, Uganda*)

Overall, providers appreciated the structured format that the SEARCH-Youth life-stage based assessments gave them for opening up their interaction with young clients by treating adolescents, young adults, and older adults as groups with distinct needs. The training associated with these assessments, augmented by additional resources according to each provider’s particular interests, led them to pick up on aspects of the young person’s life that might have caused depressive symptoms and allowed the providers to better link them to social support and mental health resources. The result was greater confidence on the part of AYAH in their providers. As one remarked, *“I am now planning on how I will take care of my child and continue taking my ARVs. Whenever I have problems, I call my provider N. and he counsels me”.* (Female, 17 y.o., intervention arm, Uganda).

## 4. Discussion

Interventions that reduce the burden of depression in AYAH globally are urgently needed [1,39,40]. In this trial of 15–24-year-olds with HIV in rural Uganda and Kenya, we found that SEARCH-Youth, a multi-level health system intervention that increased virological suppression, also reduced the prevalence of any depressive symptoms by 28%. The largest effect was among participants re-engaging with HIV care, and similar patterns of impact were observed for mild and moderate to severe depression. The qualitative in-depth interviews with AYAH, family members, and providers supported the statistical findings and shed further light on the mechanisms of intervention action.

We postulate that the SEARCH-Youth intervention’s life-stage based assessment tool, with provider training on how to use it, played a key role in reducing symptoms of depression. The life-stage based assessment tool was designed to aid providers and AYAH in identifying recent events that might affect adherence to and engagement with care. These same events could also lead to depression. At trial baseline, we found that alcohol use and having two or more significant life events were associated with low HIV treatment uptake and viral non-suppression [41]. In support of this potential mechanism, our predictor analysis found that having experienced any recent and significant life events was associated with 4.2 times higher odds of having at least mild depression. In particular, physical sickness, recent childbirth or pregnancy, and family death were predictive of at least mild depression.

Discussions of challenging issues and events could encourage participants to make changes to avoid or minimize the impact of these events. Additionally, information elicited from the discussion could also prompt the provider to link the participant or their family to counseling services. Individual counseling and family-support services have both been shown to improve both HIV and mental health outcomes among AYAH [1,42]. It is likely that the SEARCH-Youth life-stage based assessment tool worked in ways similar to “problem-focused” interventions, such as the Problem-Solving Therapy and Friendship Bench delivered by lay health workers in Zimbabwe [28,43,44].

It is also possible that the SEARCH-Youth intervention reduced symptoms of depression by helping participants build a stronger social support system. Indeed, feeling supported was associated with significantly lower odds of symptoms of at least mild depression. The life-stage based assessment tool prompted providers to ask participants if they feel supported and by whom. This line of questioning could have helped participants identify support resources and conserve or develop them further. Fewer participants reported “not feeling supported” in the intervention arm than in the control arm (2.6% vs. 5.6%). Other studies have also shown that sources of support shift during the adolescent transition to adulthood and that adolescents benefit from stability and guidance during this time [13,45].

Our results highlighted the critical role of the patient–provider interaction and the importance of trust. Unsatisfactory relationships between patients and providers, stemming from low mental health literacy among healthcare workers, have been associated with mental health challenges among AYAH in sub-Saharan Africa [1,46,47]. In our study, providers remarked on how the SEARCH-Youth training taught them to interact differently with AYAH, giving the youth time, assuring them of confidentiality, and approaching them with greater emotional attention. Other studies have highlighted how patient-centered communication to establish rapport and adopting a non-judgmental attitude affects ART adherence and depression among AYAH. Alleviating poor patient–provider interactions, historically associated with depressive symptoms among AYAH in sub-Saharan Africa, is an additional mechanism by which the intervention had an effect. However, our qualitative data suggest that positive clinical encounters with providers could also become a new support system for AYAH. Discussions allowed participants to open up and talk about challenges, gave providers an opportunity to advise them, and helped participants feel happier with their lives during a period of shifting sources of support.

We found that a history or fear of physical harm and feeling pressured to have sexual activity were strong predictors of at least mild depression in our cohort. Other studies have found strong relationships between depression and sexual and physical abuse in Africa [48,49]. In our study, fewer intervention participants reported a history (or threat) of physical harm or feeling pressured to perform sexual activity, as compared to control arm participants. While it is possible that the differences between the arms occurred by chance, our intervention might have helped AYAH navigate difficult relationships by talking through the nature of threats and identifying social support. A large evidence base suggests that social support can help mitigate harm from sexual violence in adolescent girls and young women.

Our qualitative data also suggest that the interaction with providers increased confidence and a sense of self in AYAH. In other studies, having a strong sense of self not tied to HIV status has been shown to be a protective factor against abuse. Through the SEARCH-Youth assessments, providers helped participants attenuate self-hate (internalized stigma) and develop a stronger sense of self. The facilitated discussions might also have helped impart more self-assured communication skills among participants. The process of planning and successfully navigating challenges might have increased participants’ sense of self-efficacy, which studies have shown improves mental health [50,51,52]. Additionally, the connection with the providers may have alleviated the perception of rejection by others, which has previously been associated with a reduction in senses of loneliness and depression. Having a future orientation is a notable protective factor that promotes building supportive caregiving contexts [53,54] Other studies have observed that college-age women from African backgrounds in the United States sometimes delay discussing violence from intimate partners with friends for fear they will be pressured to leave the relationship, which in their cultural contexts means jeopardizing a future marriage. If these cultural patterns also hold for AYAH in Kenya and Uganda, it may be that opening up new future trajectories offers alternatives to avoiding or escaping abusive relationships.

### 4.1. Limitations

There are several limitations to our study. First, by administering the PHQ-9 survey cross-sectionally, we were unable to evaluate changes over time in depressive symptoms. However, given randomization, the baseline prevalence of depressive symptoms should have been balanced by arm and not biased our results. Second, when considering the generalizability of our results, it is important to note that during the study period, several other programs supporting AYAH were operating in the study communities. These are summarized in Table A4 (Appendix A) and provided psychosocial, nutritional, family, and material support to AYAH. In the qualitative studies, these organizations were mentioned by providers but not by young participants or their families. Therefore, the effect of these organizations was difficult to evaluate from the participants’ perspective. However, we would not expect them to have a differential effect on the intervention or control communities. Indeed, the significant effect of the SEARCH-Youth intervention on the prevalence of any depressive symptoms suggests the added value of our multi-level and multi-component intervention on top of existing programs. Third, the prevalence of depressive symptoms among the SEARCH-Youth participants was lower than that reported in other studies [2,46,55]. It is likely that some underreporting occurred due to participants not wanting to disappoint staff or experience shame; the underreporting of gender-based violence in adolescents is particularly common [56,57]. Nonetheless, mild depression symptoms were more common and had the same risk factors as depressive disorders with the same effect on quality of life. Finally, we cannot rule out the possibility that after 3 years of follow-up, participants with severe depression had disengaged from this study.

### 4.2. Potential Future Directions

The SEARCH-Youth intervention is being adapted and studied in community implementation projects. It is also possible that the SEARCH-Youth life-stage counseling tool could help bridge the transition between pediatric and adult HIV care, a period with high rates of attrition [58].

## 5. Conclusions

The SEARCH-Youth intervention reduced the prevalence of depressive symptoms in a cohort of AYAH in rural Uganda and Kenya. The SEARCH-Youth life-stage based assessment appears to have played a key role in improving mental health by improving patient–provider interactions, facilitating trust, augmenting existing support systems, helping AYAH identify new sources of support, and linking them to additional external resources as appropriate.

## Figures and Tables

**Table 1:** Characteristics of depression study participants at time of enrollment in the SEARCH-Youth trial.

	Depression Survey			Qualitative Study
	Control (N = 572)	Intervention (N = 662)	Overall (N = 1234)	Overall (N = 113 ^#^)
**Age**				
Median [min., max.]	21.0 [15.0, 24.0]	21.0 [15.0, 24.0]	21.0 [15.0, 24.0]	21.0 [15.0, 24.0]
**Sex**				
Female	451 (78.8%)	539 (81.4%)	990 (80.2%)	70 (63.6%)
**Country**				
Kenya	258 (45.1%)	313 (47.3%)	571 (46.3%)	64 (58.2%)
Uganda	314 (54.9%)	349 (52.7%)	663 (53.7%)	46 (41.8%)
**Marital status**				
Single	230 (40.2%)	297 (44.9%)	527 (42.7%)	45 (40.9%)
Married, monogamous	239 (41.8%)	258 (39.0%)	497 (40.3%)	46 (41.8%)
Widowed	4 (0.7%)	4 (0.6%)	8 (0.6%)	2 (1.8%)
Divorced	59 (10.3%)	67 (10.1%)	126 (10.2%)	10 (9.1%)
Married, polygamous	40 (7.0%)	36 (5.4%)	76 (6.2%)	7 (6.4%)
**Education**				
No school	22 (3.8%)	25 (3.8%)	47 (3.8%)	0 (0%)
Primary school	376 (65.7%)	420 (63.4%)	796 (64.5%)	81 (73.6%)
Secondary school	139 (24.3%)	172 (26.0%)	311 (25.2%)	26 (23.6%)
Tertiary school	35 (6.1%)	45 (6.8%)	80 (6.5%)	3 (2.7%)
**Employment status**				
Employed	227 (39.7%)	221 (33.4%)	448 (36.3%)	36 (32.7%)
In school	127 (22.2%)	155 (23.4%)	282 (22.9%)	25 (22.7%)
Unemployed	218 (38.1%)	286 (43.2%)	504 (40.8%)	49 (44.5%)
**Baseline care status**				
New to care ^	136 (23.8%)	188 (28.4%)	324 (26.3%)	58 (52.7%)
Engaged in care *	425 (74.3%)	444 (67.1%)	869 (70.4%)	42 (38.2%)
Re-engaging in care ^##^	11 (1.9%)	30 (4.5%)	41 (3.3%)	10 (9.1%)

Values are n (% of subcategory) unless otherwise noted. ^#^ Baseline characteristics are missing for three participants in the intervention arm. ^ Started ART within the prior 6 months or at enrolment. * Started ART more than 6 months ago and had a clinic visit in the prior 6 months. ^##^ Started ART more than 6 months ago and did not have a clinic visit in the prior 6 months.

**Table 2:** Recent major life events.

	Control	Intervention	Overall
	(N = 572)	(N = 662)	(N = 1234)
**Major life events:**			
Had events in prior 3 months	232 (40.6%)	219 (33.1%)	451 (36.5%)
Changed residence	99 (17.3%)	72 (10.9%)	171 (13.9%)
Changed employment	62 (10.8%)	62 (9.4%)	124 (10.0%)
Sickness	61 (10.7%)	44 (6.6%)	105 (8.5%)
New partner	34 (5.9%)	26 (3.9%)	60 (4.9%)
Birth in last month or pregnant	24 (4.2%)	27 (4.1%)	51 (4.1%)
Family death	23 (4.0%)	27 (4.1%)	50 (4.1%)
Family strife	14 (2.4%)	7 (1.1%)	21 (1.7%)
Relationship strife	14 (2.4%)	25 (3.8%)	39 (3.2%)
Incarceration	1 (0.2%)	2 (0.3%)	3 (0.2%)

**Table 3:** Social factors and substance use.

	Control	Intervention	Overall
	(N = 572)	(N = 662)	(N = 1234)
**Social pressures and supports:**			
Does not feel supported	32 (5.6%)	17 (2.6%)	49 (4.0%)
Feels threatened	28 (4.9%)	20 (3.0%)	48 (3.9%)
Feels pressured to have sex	13 (2.3%)	3 (0.5%)	16 (1.3%)
** Alcohol use: **			
Does not drink alcohol	436 (76.2%)	533 (80.5%)	969 (78.5%)
Drinks alcohol	110 (19.2%)	129 (19.5%)	239 (19.4%)
Missing	26 (4.5%)	0 (0%)	26 (2.1%)
** Other Substance use: **			
Uses other (e.g., marijuana)	6 (1.0%)	12 (1.8%)	18 (1.5%)

## Data Availability

The raw data supporting the conclusions of this article will be made available by the authors on request.

## Appendix A

**Table A1:** Depression survey.

Prompt: Over the last 2 weeks, how often have you been bothered by any of the following problems?				
	**Not** **At All**	**Several** **Days**	**More than** **Half the Days**	**Nearly** **Every Day**
	**0**	**1**	**2**	**3**
1. Little interest or pleasure in doing things	□	□	□	□
2. Feeling down, depressed, or hopeless	□	□	□	□
3. Trouble falling/staying asleep, sleeping too much	□	□	□	□
4. Feeling tired or having little energy	□	□	□	□
5. Poor appetite or overeating	□	□	□	□
6. Feeling bad about yourself—or that you are a failure or have let yourself or your family down.	□	□	□	□
7. Trouble concentrating on things, such as reading the newspaper or watching television.	□	□	□	□
8. Moving or speaking so slowly that other people could have noticed. Or the opposite—being so fidgety or restless that you have been moving around a lot more than usual.	□	□	□	□
9. Thoughts that you would be better off dead or of hurting yourself in some way.	□	□	□	□
If an individual scores 1 or more on question 9: a. Do you currently have these thoughts? b. Have you made a plan to hurt yourself in some way?	Yes Yes	No No		
If an individual scored 2 or 3 on any 5 symptoms: a. Are you able to carry out your day-to-day activities? b. Are you engaging with people the way you normally do? c. Or have you stopped doing things (e.g., so tired or worried, that you don’t get anything done or interact with anyone) d. Is your functioning (social, work, home) impaired?	Yes Yes Yes Yes	No No No No		
**Life Events**				
**Have you visited any other clinic services in the last 3 months?** 1: Reproductive health services 2: Mental health services 3: Peer support 4: Facilitated disclosure services 5: Other 99: Did not access other clinic service **Do any of the following apply to you?** 1: Currently pregnant 2. Gave birth in the last 1 month 3. Suffered pregnancy loss 4. None of the above **Any recent major life events in the last 3 months?** 1: Start or stop school or employment 2: Change in residence 3: Divorce, separation or relationship strife 4: New sexual partner 5: Family death 6: Sickness 7: Incarceration 8: Family strife 9: Birth or pregnancy -8: Refuse 99: None of the Above	**Describe details about major recent life events** **Do you feel supported by people around you?** 1: Yes 0: No [If yes] **Who is your support?** **Does anyone ever hurt or threaten you?** 1: Yes 0: No 8: Refuse **If yes, who?** Parent Partner Other None **Do you feel pressured to have sexual activity?** Yes No Refuse			

**Table A2:** Baseline characteristics of participants in qualitative study.

	Control (N = 34)	Intervention (N = 79) *^#^*	Overall (N = 113) *^#^*
**Age**			
Median [min., max.]	20.5 [15.0, 24.0]	21.0 [15.0, 24.0]	21.0 [15.0, 24.0]
**Sex**			
Female	18 (52.9%)	52 (68.4%)	70 (63.6%)
**Country**			
Kenya	19 (55.9%)	45 (59.2%)	64 (58.2%)
Uganda	15 (44.1%)	31 (40.8%)	46 (41.8%)
**Marital status**			
Single, never married	16 (47.1%)	29 (38.2%)	45 (40.9%)
Married, monogamous	14 (41.2%)	32 (42.1%)	46 (41.8%)
Widowed	0 (0%)	2 (2.6%)	2 (1.8%)
Divorced	2 (5.9%)	8 (10.5%)	10 (9.1%)
Married, polygamous	2 (5.9%)	5 (6.6%)	7 (6.4%)
**Education**			
No school	0 (0%)	0 (0%)	0 (0%)
Primary school	26 (76.5%)	55 (72.4%)	81 (73.6%)
Secondary school	7 (20.6%)	19 (25.0%)	26 (23.6%)
Tertiary school	1 (2.9%)	2 (2.6%)	3 (2.7%)
**Employment status**			
Employed	10 (29.4%)	26 (34.2%)	36 (32.7%)
In school	9 (26.5%)	16 (21.1%)	25 (22.7%)
Unemployed	15 (44.1%)	34 (44.7%)	49 (44.5%)
**Number of children**			
Median [min., max.]	0 [0, 3.00]	1.00 [0, 3.00]	0 [0, 3.00]
**Baseline care status**			
New to care ^	19 (55.9%)	39 (51.3%)	58 (52.7%)
Engaged in care *	13 (38.2%)	29 (38.2%)	42 (38.2%)
Re-engaging in care ^#^	2 (5.9%)	8 (10.5%)	10 (9.1%)

Values are n (% of subcategory) unless otherwise noted. ^#^ Data are not available for 3 participants in the intervention arm. ^ Started ART within the prior 6 months or at enrolment. * Started ART more than 6 months ago and had a clinic visit in prior 6 months. ^#^ Started ART more than 6 months prior and did not have a clinic visit the prior 6 months.

**Table A3:** PHQ-9 scores.

	Control	Intervention	Overall
	(N = 572)	(N = 662)	(N = 1234)
** Total PHQ-9 score **			
Mean (SD)	2.11 (2.33)	1.16 (1.73)	1.60 (2.08)
Median [min., max.]	1.00 [0, 15.0]	1.00 [0, 11.0]	1.00 [0, 15.0]
** Score >= 1 **			
Score = 0	154 (26.9%)	313 (47.3%)	467 (37.8%)
Score >= 1	418 (73.1%)	349 (52.7%)	767 (62.2%)
** Score >= 5 **			
Score < 5	512 (89.5%)	631 (95.3%)	1143 (92.6%)
Score >= 5	60 (10.5%)	31 (4.7%)	91 (7.4%)
** Score >= 10 **			
Score < 10	561 (98.1%)	656 (99.1%)	1217 (98.6%)
Score >= 10	11 (1.9%)	6 (0.9%)	17 (1.4%)
** PHQ-1: Little interest or pleasure in doing things **			
Not at all	442 (77.3%)	601 (90.8%)	1043 (84.5%)
Several days	113 (19.8%)	55 (8.3%)	168 (13.6%)
More than half the days	12 (2.1%)	5 (0.8%)	17 (1.4%)
Nearly every day	5 (0.9%)	1 (0.2%)	6 (0.5%)
** PHQ-2: Feeling down, depressed, or hopeless **			
Not at all	425 (74.3%)	596 (90.0%)	1021 (82.7%)
Several days	129 (22.6%)	54 (8.2%)	183 (14.8%)
More than half the days	15 (2.6%)	11 (1.7%)	26 (2.1%)
Nearly every day	3 (0.5%)	1 (0.2%)	4 (0.3%)
** PHQ-3: Trouble falling/staying asleep, sleeping too much **			
Not at all	385 (67.3%)	552 (83.4%)	937 (75.9%)
Several days	164 (28.7%)	98 (14.8%)	262 (21.2%)
More than half the days	19 (3.3%)	12 (1.8%)	31 (2.5%)
Nearly every day	4 (0.7%)	0 (0%)	4 (0.3%)
** PHQ-4: Feeling tired or having little energy **			
Not at all	361 (63.1%)	485 (73.3%)	846 (68.6%)
Several days	181 (31.6%)	153 (23.1%)	334 (27.1%)
More than half the days	27 (4.7%)	21 (3.2%)	48 (3.9%)
Nearly every day	3 (0.5%)	3 (0.5%)	6 (0.5%)
** PHQ-5: Poor appetite or overeating **			
Not at all	410 (71.7%)	519 (78.4%)	929 (75.3%)
Several days	142 (24.8%)	126 (19.0%)	268 (21.7%)
More than half the days	17 (3.0%)	10 (1.5%)	27 (2.2%)
Nearly every day	3 (0.5%)	7 (1.1%)	10 (0.8%)
** PHQ-6: Feeling bad about yourself **			
Not at all	494 (86.4%)	614 (92.7%)	1108 (89.8%)
Several days	70 (12.2%)	44 (6.6%)	114 (9.2%)
More than half the days	6 (1.0%)	4 (0.6%)	10 (0.8%)
Nearly every day	2 (0.3%)	0 (0%)	2 (0.2%)
** PHQ-7: Trouble concentrating on things **			
Not at all	496 (86.7%)	622 (94.0%)	1118 (90.6%)
Several days	66 (11.5%)	38 (5.7%)	104 (8.4%)
More than half the days	9 (1.6%)	2 (0.3%)	11 (0.9%)
Nearly every day	1 (0.2%)	0 (0%)	1 (0.1%)
** PHQ-8: Moving or speaking slowly **			
Not at all	530 (92.7%)	641 (96.8%)	1171 (94.9%)
Several days	38 (6.6%)	19 (2.9%)	57 (4.6%)
More than half the days	3 (0.5%)	2 (0.3%)	5 (0.4%)
Nearly every day	1 (0.2%)	0 (0%)	1 (0.1%)
** PHQ-9: Thoughts that you would be better off dead **			
Not at all	555 (97.0%)	652 (98.5%)	1207 (97.8%)
Several days	11 (1.9%)	7 (1.1%)	18 (1.5%)
More than half the days	6 (1.0%)	2 (0.3%)	8 (0.6%)
Nearly every day	0 (0%)	1 (0.2%)	1 (0.1%)

**Table A4:** Regional programs in Kenya and Uganda.

**Program** **Name**	**Description**
YAPS	Young Adolescent Peer Support (YAPS), a youth peer support program
PATA	Pediatric and adolescent Treatment Africa, which supports children and adolescents to start and remain on ART
NAYA	Provides psychosocial support to increase resilience among young people with HIV in Kenya
Blue Cross	An alcohol and substance abuse and mental health support program
Ariel Club	A program of the Elizabeth Glazer foundation (EGPAF) targeting children and adolescents up to 19 years old with group psychosocial support at a health facility
TASO	The AIDS Support Organization, which places trained counsellors in public health facilities for adherence support
TPO	Transcultural Psychosocial Organization, which targets the mental health of youth with viral non-suppression in Uganda, offering home visits and food
Mwendo	Supports orphans and vulnerable children with physical and psychosocial needs
